# Insights to a Cure: Unique Controller Phenotypes in the Rotterdam HIV-2 Cohort

**DOI:** 10.1093/ofid/ofaf336

**Published:** 2025-06-18

**Authors:** Kathryn S Hensley, Rob A Gruters, Els van Nood, Mariana de Mendonça Melo, Ronald J Overmars, Alicja U Górska, Casper Rokx, Cynthia Lungu, David A M C van de Vijver, Thibault Mesplède, Jeroen J A van Kampen

**Affiliations:** Department of Internal Medicine, Section Infectious Diseases, Erasmus University Medical Centre, Rotterdam, Netherlands; Department of Medical Microbiology and Infectious Diseases, Erasmus University Medical Centre, Rotterdam, Netherlands; Department of Viroscience, Erasmus University Medical Centre, Rotterdam, Netherlands; Department of Internal Medicine, Section Infectious Diseases, Erasmus University Medical Centre, Rotterdam, Netherlands; Department of Medical Microbiology and Infectious Diseases, Erasmus University Medical Centre, Rotterdam, Netherlands; Department of Internal Medicine, Section Infectious Diseases, Erasmus University Medical Centre, Rotterdam, Netherlands; Department of Medical Microbiology and Infectious Diseases, Erasmus University Medical Centre, Rotterdam, Netherlands; Department of Viroscience, Erasmus University Medical Centre, Rotterdam, Netherlands; Department of Medical Microbiology and Infectious Diseases, Erasmus University Medical Centre, Rotterdam, Netherlands; Department of Viroscience, Erasmus University Medical Centre, Rotterdam, Netherlands; Department of Internal Medicine, Section Infectious Diseases, Erasmus University Medical Centre, Rotterdam, Netherlands; Department of Medical Microbiology and Infectious Diseases, Erasmus University Medical Centre, Rotterdam, Netherlands; Department of Viroscience, Erasmus University Medical Centre, Rotterdam, Netherlands; Department of Viroscience, Erasmus University Medical Centre, Rotterdam, Netherlands; Department of Viroscience, Erasmus University Medical Centre, Rotterdam, Netherlands; Department of Viroscience, Erasmus University Medical Centre, Rotterdam, Netherlands

**Keywords:** elite controllers, HIV-2, HIV-2 elite control, nonviremic progressor, recontroller

## Abstract

**Background:**

HIV-2, although less common than HIV-1, exhibits a higher proportion of elite controllers (ECs), who can suppress HIV without antiretroviral therapy (ART), a phenomenon rarely observed in HIV-1. Studying ECs could yield insights into viral control mechanisms and potentially lead to a cure.

**Methods:**

We retrospectively characterized a cohort of people with HIV-2 who received care at the Erasmus University Medical Center, Rotterdam, Netherlands. The aim was to identify categories of ECs based on plasma viral loads, CD4+ T-cell count, and responses to ART.

**Results:**

Between 1989 and 2023, 52 people with HIV-2 were included, primarily of West African origin (80.8%). Follow-up ranged from <1 to 32 years (median, 16 years). Seven participants were lost to follow-up (13.5.%), and 18 participants died (34.6%), 7 before ART availability due to AIDS. The remaining 40 participants were included in the detailed analysis. Thirteen were ECs with CD4+ T cells >350 cells/mm^3^ and viral loads <200 copies/mL without use of ART. Four participants progressed to CD4+ T cells <350 cells/mm^3^ without symptoms of HIV despite undetectable viral loads (nonviremic progressors). Three individuals demonstrated EC status for at least 5 years but lost viral and immunologic control. Nineteen participants exhibited a classical phenotype of viremic progression. Five participants had HIV-1 and HIV-2. Finally, 1 participant had a unique phenotype with loss of control with an unexplained rebound in viremia, followed by resuppression without ART for >10 years (recontroller).

**Conclusions:**

These data highlight relevant trajectories among ECs. Understanding the underlying mechanisms can inform decisions on treatment and contribute to finding a cure for all people with HIV.

HIV-2 is lesser known and less prevalent than HIV-1 [1]. Interestingly, as compared with HIV-1, HIV-2 infections yield a higher proportion of elite controllers (ECs): individuals who can control plasma viral load without antiretroviral therapy (ART) [2]. Also more frequent within HIV-2 than HIV-1 are long-term nonprogressors (LTNPs): those who remain asymptomatic and maintain high CD4+ T-cell counts despite low-level viremia in the absence of ART. Together, these individuals are 10- to 40-fold more frequent among people with HIV-2 [2]. The reasons for this difference are not yet fully understood. Amid the global pursuit of an HIV cure, insights learned from HIV-2 ECs hold promise [3]. Studying the clinical trajectories, viral characteristics, immune responses, and genetic factors associated with elite control in HIV-2 will help to extrapolate valuable lessons applicable to HIV-1 cure research [4, 5].

HIV-2 is prevalent in limited regions, particularly West Africa, where it can be responsible for a considerable proportion of HIV infections [6–8]. In Europe, due to colonial history and migratory patterns, the largest populations of people with HIV-2 are in Portugal and France [2, 9]. In the Netherlands in 2022, only 4% of documented people with HIV were diagnosed with HIV-2 [10]. The Rotterdam cohort, first described in 1996, consists mainly of West African immigrants due to the large port of Rotterdam and encompasses almost a third of all people with HIV-2 in the Netherlands [11].

In this article, we build on the work of several European HIV-2 cohorts, which have provided important insights into the clinical course, treatment response, and virologic characteristics of HIV-2 [2, 12–14]. We give an update on the Rotterdam cohort of people with HIV-2 [5, 11, 15, 16], focusing on clinical and virologic data from decades of follow-up. We identify several categories of people who differ in clinical and immunologic progression and viral loads, some of whom display phenotypes rarely observed within HIV-1. This overview underscores the benefits of comprehensively characterizing cohorts of people with HIV-2, particularly in their long-term natural disease evolution, but also the need for more epidemiologic, clinical, and virologic data. Such data from HIV-2 could uncover curative strategies for all people with HIV.

## MATERIALS AND METHODS

### Study Setting and Population

This retrospective cohort consists of adults diagnosed with HIV-2 at the Erasmus University Medical Centre (Erasmus MC), Rotterdam, the Netherlands. All people diagnosed with HIV-2 were eligible for inclusion. Participants were included after informed consent. Data were collected from electronic data record files and included sex assigned at birth, mode of HIV acquisition, country of birth, date of HIV diagnosis, AIDS-defining diseases, date and cause of death (if applicable), use of ART, CD4+ T-cell counts, HIV-2 and HIV-1 plasma viral load, and hepatitis B and C status. Participants’ deaths were recorded as being due to HIV if the cause of death was an AIDS-defining condition. Participants were deemed lost to follow-up when they were not in care per their own choice or because they could not be reached. In routine practice, viral load monitoring and/or CD4+ T-cell count occurred twice a year. We categorized participants based on HIV-2 plasma viral load, CD4+ T-cell counts, and use of ART for the total duration of follow-up (ie, a single patient was assigned to a single clinical phenotype; Table 1).

**Table 1.** Clinical Phenotypes of HIV-2

Name	HIV-2 Viral Load	CD4+ T-Cell Count	Antiretroviral Therapy
Elite controllers	<200 copies/mL; 1 blip >200 copies/mL allowed^a^	>350 cells/mm^3^; 1 dip <350 cells/mm^3^ allowed	No, except exclusively during pregnancy^b^
Loss of control			
At least 5 y of elite controller phenotype, then loss of control	≥2 subsequent measurements >200 copies/mL^c^	<350 cells/mm^3^ or AIDS-defining conditions	Yes
Progressors			
Nonviremic	<200 copies/mL; 1 blip >200 copies/mL allowed^a^	<350 cells/mm^3^ or AIDS-defining conditions	Yes
Viremic	≥2 subsequent measurements >200 copies/mL^c^	<350 cells/mm^3^ or AIDS-defining conditions	Yes

Definitions of the clinical phenotypes of HIV-2 used in this study.

^a^For HIV-2 viral load measurements performed before 2002, <500 copies/mL and no blips allowed.

^b^Not a recommendation.

^c^For HIV-2 viral load measurements performed before 2002, ≥2 subsequent measurements >500 copies/mL.

### Patient Consent Statement

Cohort management was in accordance with the principles of the Declaration of Helsinki, good clinical practice guidelines, and the Dutch Medical Research Involving Human Subjects Act. Ethical approval was given by the Medical Ethics Review Committee from the Erasmus MC: METC-2000-221 (HIV-2 sample collection), METC-2022-060 (HIV-1/2 biobank), and METC-2012-583 (ex vivo study, NCT05215704). All participants in care after 2001 consented to use of their data in the AIDS Therapy Evaluation in the Netherlands cohort, except for participants deceased or lost to follow-up >20 years ago.

### Viral Load Assays

Since the start of inclusion, 2 HIV-2 RNA viral load assays have been used with different lower limits of detection. The initial HIV-2 RNA viral load assay had a limit of detection of 500 copies/mL, which improved to 50 copies/mL in 2002. To simplify, we display a limit of detection for 50 copies/mL in the figures and indicate if it was 500 copies/mL with an asterisk. The HIV-2 viral assays have been used as described before [17, 18]. Briefly, designs of the primers and probes for groups A and B were based on the HIV-2 group A and B consensus sequence per Primer Express software (PE Biosystems). Primers were synthesized by Isogen Bioscience BV. Probes were labeled with the fluorochrome FAM at the 5′ end and the quencher TAMRA at the 3′ end and synthesized by PE Biosystem. RNA was isolated from plasma via the MagNA Pure LC Total Nucleic Acid Large Volume Kit (Roche Diagnostics).

### Statistical Analysis

Descriptive data are presented as number (percentage) or median (IQR). No predefined hypothesis was formulated at the cohort start. We calculated the change in CD4+ T-cell counts with linear regressions, where a negative slope was considered a decline and a positive slope an increase. Slopes are shown with 95% CIs. Data were collected in Excel and analyzed by Prism version 9 (GraphPad).

## RESULTS

### Baseline Characteristics

Between 1989 and 2023, the Rotterdam Erasmus MC HIV-2 cohort included 52 people with HIV-2 (Table 2). Most participants were born in West Africa (80.8%), of which the majority was from Cape Verde, corresponding with the large community of migrants from Cape Verde living in Rotterdam. Follow-up ranged from <1 to 32 years with a median 16 years. Median CD4+ T-cell count at diagnosis was 240 cells/mm^3^ (IQR, 80–740). Most participants presented with a Centers for Disease Control and Prevention (CDC) category A (59.6%). All hepatitis B virus infections occurred before participants were diagnosed; therefore, they had already recovered or started therapy with ART. Seven participants were lost to follow-up (13.5.%) and 18 died (34.6%). Seven people died before combination ART was available, as well as before HIV-2 viral load assays were available. It is thus impossible to characterize them in the defined categories, and they have been classified in a separate category (pre-ART). We categorized the other people in this cohort in 4 categories based on level of control: ECs (n = 13), participants with HIV-2 loss of control after EC status (n = 3), nonviremic progressors (n = 4), and viremic progressors (n = 19). There were 5 participants with HIV-1 and HIV-2 dual infection. Finally, 1 participant exhibited loss of control but was able to control this without ART, a phenotype that defied categorization and has been described separately (recontroller). Each participant was categorized by total duration of follow-up, and group assignment did not change over time.

**Table 2.** Characteristics of the Erasmus MC HIV-2 Cohort

	Elite Controller (n = 13)	Recontroller (n = 1)	Loss of Control (n = 3)	Nonviremic Progressor (n = 4)	Viremic Progressor (n = 19)	HIV-1 + HIV-2 (n = 5)	Pre-ART (n = 7)	Total (n = 52)
Sex assigned at birth								
Male	1 (7.7)	1 (100.0)	2 (66.7)	2 (50.0)	11 (57.9)	4 (80.0)	4 (57.1)	25 (48.1)
Female	12 (92.3)	0 (0.0)	1 (33.3)	2 (50.0)	8 (42.1)	1 (20.0)	3 (42.9)	27 (51.9)
Age at diagnosis, y	36 (33–38)	54	29 (26–37)	51 (40–58)	45 (40.5–51)	47 (39–49)	35 (34–39)	40 (34–49)
Follow-up, y^a^	17 (14–20)	18	27 (21–29)	14 (11–17)	21 (8–24)	14 (11–14)	2 (2–4)	16 (5–22)
Region of birth								
West Africa–Cape Verde	6 (46.2)	0 (0.0)	2 (66.7)	1 (25.0)	14 (73.7)	2 (40.0)	5 (71.4)	30 (57.7)
West Africa–other	6 (46.2)	0 (0.0)	0 (0.0)	2 (50.0)	2 (10.5)	1 (20.0)	1 (14.3)	12 (23.1)
Europe	0 (0.0)	1 (100.0)	1 (33.3)	0 (0.0)	3 (15.8)	2 (40.0)	1 (14.3)	8 (15.4)
Other	1 (7.7)	0 (0.0)	0 (0.0)	1 (25.0)	0 (0.0)	0 (0.0)	0 (0.0)	2 (3.8)
Mode of transmission								
Heterosexual male to female	7 (53.8)	0 (0.0)	1 (33.3)	1 (25.0)	6 (31.6)	0 (0.0)	2 (28.6)	17 (32.7)
Heterosexual female to male	0 (0.0)	1 (100.0)	1 (33.3)	1 (25.0)	7 (36.8)	1 (20.0)	3 (42.9)	14 (26.9)
Men who have sex with men	0 (0.0)	0 (0.0)	1 (33.3)	0 (0.0)	0 (0.0)	1 (20.0)	0 (0.0)	2 (3.8)
Blood products/transfusion	1 (7.7)	0 (0.0)	0 (0.0)	0 (0.0)	1 (5.3)	0 (0.0)	0 (0.0)	2 (3.8)
ND	5 (38.5)	0 (0.0)	0 (0.0)	2 (50.0)	5 (26.3)	3 (60.0)	2 (28.6)	17 (32.7)
CDC stage at diagnosis								
A	12 (92.3)	1 (100.0)	3 (100.0)	4 (100.0)	8 (42.1)	3 (60.0)	0 (0.0)	31 (59.6)
B	0 (0.0)	0 (0.0)	0 (0.0)	0 (0.0)	3 (15.8)	0 (0.0)	3 (42.9)	6 (11.5)
C	0 (0.0)	0 (0.0)	0 (0.0)	0 (0.0)	6 (31.6)	2 (40.0)	3 (42.9)	11 (21.2)
ND	1 (7.7)	0 (0.0)	0 (0.0)	0 (0.0)	2 (10.5)	0 (0.0)	1 (14.3)	4 (7.7)
T cells at diagnosis, cells/mm^3^								
CD4+	750 (670–850)	980	690 (650–730)	305 (205–373)	110 (70–190)	50 (30–60)	140 (10–160)	240 (80–740)
CD8+	680 (470–820)	980	920 (890–950)	520 (425–718)	590 (350–940)	530 (480–720)	690 (280–780)	630 (400–900)
CD4/CD8 ratio at diagnosis	1.1 (0.8–1.7)	1.0	0.7 (0.7–0.8)	0.4 (0.3–0.5)	0.2 (0.1–0.3)	0.1 (0.1–0.1)	0.2 (0.1–0.2)	0.3 (0.1–1.0)
HIV-2 RNA, copies/mL								
Always undetectable	7 (53.8)	NA	0 (0.0)	3 (75.0)	0 (0.0)	2 (40.0)	ND	12 (23.1)
Zenith, if detectable	131 (92–318)	89 000	6800 (4275–8200)	71	475 000 (10 875–112 250)	170 000 (108 500–785 000)	ND	12 200 (2763–89 000)
HIV-1 RNA zenith, copies/mL	NA	NA	NA	NA	NA	49 000 (22 100–595 000)	NA	49 000 (22 100–595 000)
Hepatitis C coinfection								
Yes	0 (0.0)	0 (0.0)	1 (33.3)	0 (0.0)	1 (5.3)	0 (0.0)	0 (0.0)	2 (3.8)
No	12 (92.3)	1 (100.0)	2 (66.7)	4 (100.0)	16 (84. 2)	4 (80.0)	2 (28.6)	41 (78.8)
ND	1 (7.7)	0 (0.0)	0 (0.0)	0 (0.0)	2 (10.5)	1 (20.0)	5 (71.4)	9 (17.3)
Hepatitis B virus coinfection								
Yes	0 (0.0)	0 (0.0)	0 (0.0)	1 (25.0)	2 (10.5)	1 (20.0)	2 (28.6)	6 (11.5)
Cleared	9 (69.2)	0 (0.0)	1 (33.3)	2 (50.0)	10 (52.6)	3 (60.0)	1 (14.3)	26 (50.0)
No	1 (7.7)	1 (100.0)	1 (33.3)	1 (25.0)	2 (10.5)	1 (20.0)	0 (0.0)	7 (13.5)
Vaccinated	2 (15.4)	0 (0.0)	1 (33.3)	0 (0.0)	4 (21.1)	0 (0.0)	0 (0.0)	7 (13.5)
ND	1 (7.7)	0 (0.0)	0 (0.0)	0 (0.0)	1 (5.3)	0 (0.0)	4 (57.1)	6 (11.5)
Antiretroviral therapy								
No	13 (100.0)^b^	1 (100.0)	0 (0.0)	0 (0.0)	0 (0.0)	0 (0.0)	7 (100.0)	21 (40.4)
Yes	0 (0.0)	0 (0.0)	3 (100.0)	4 (100.0)	19 (100.0)	5 (100.0)	0 (0.0)	31 (59.6)
Outcome								
Deceased–not HIV related	0 (0.0)	1 (100.0)	0 (0.0)	2 (50.0)	2 (10.5)	0 (0.0)	0 (0.0)	5 (9.6)
Deceased–HIV related	0 (0.0)	0 (0.0)	0 (0.0)	0 (0.0)	2 (10.5)	1 (20.0)	7 (100.0)	10 (19.2)
Deceased–unknown	0 (0.0)	0 (0.0)	0 (0.0)	0 (0.0)	1 (5.3)	2 (40.0)	0 (0.0)	3 (5.8)
Alive	9 (69.2)	0 (0.0)	3 (100.0)	2 (50.0)	11 (57.9)	2 (40.0)	0 (0.0)	27 (51.9)
Lost to follow-up	4 (30.8)	0 (0.0)	0 (0.0)	0 (0.0)	3 (15.8)	0 (0.0)	0 (0.0)	7 (13.5)
Age at death, y	NA	72	NA	74	58 (51–67)	51 (47–56)	36 (36–43)	50 (40–62)

Data are No. (%) or median (IQR).

Abbreviations: ART, antiretroviral therapy; CDC, Centers for Disease Control and Prevention; Erasmus MC, Erasmus University Medical Centre; NA, not applicable; ND, no data.

^a^Years between year of diagnosis and year of last CD4 count or viral load, year of death, or year of loss to follow-up.

^b^Three female elite controllers were treated temporarily with ART during pregnancy.

### Elite Controllers

**Figure 1.**
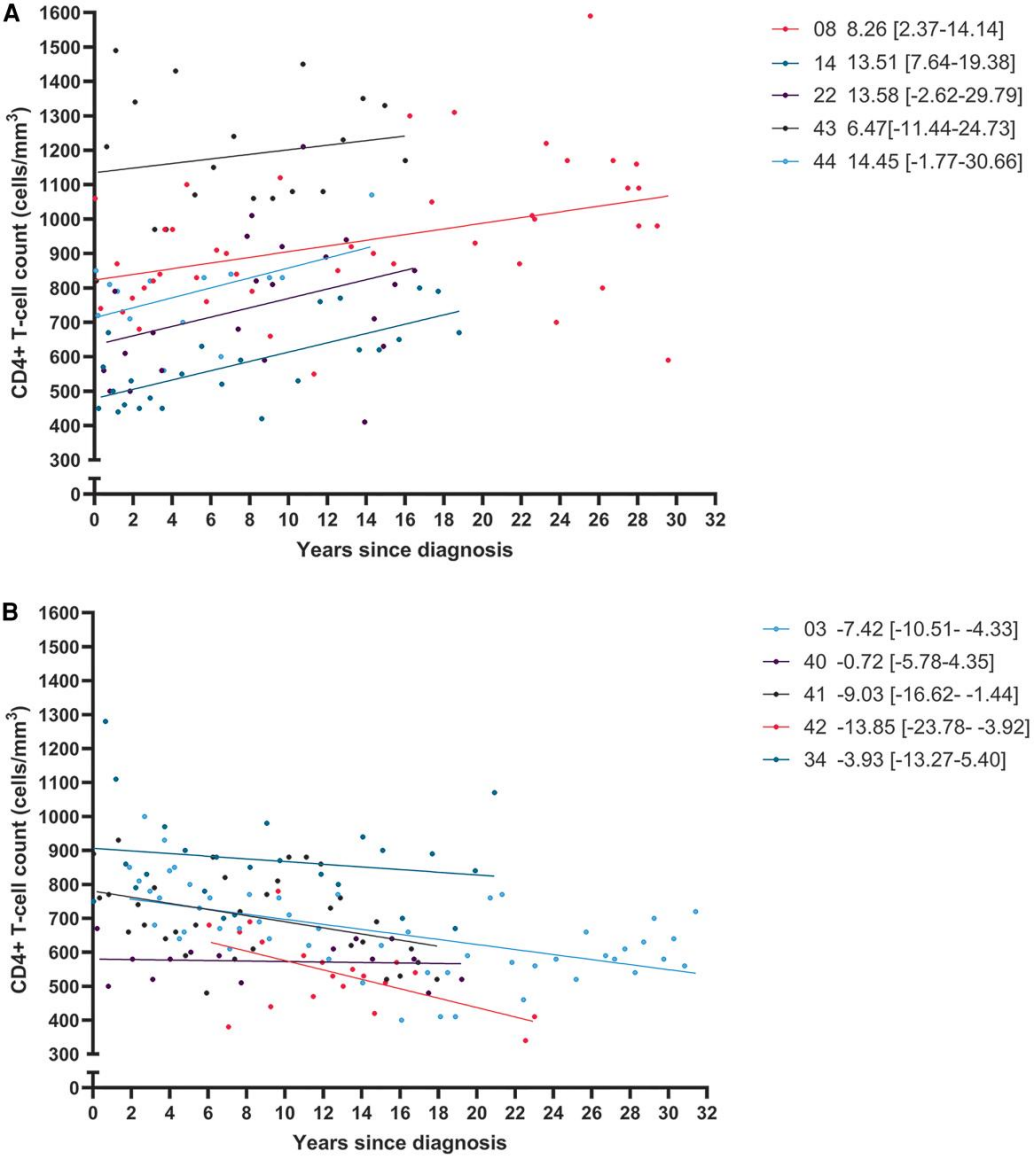
CD4+ T-cell counts in HIV-2 elite controllers of the Rotterdam HIV-2 cohort in care for >10 years. The y-axis shows the CD4+ T-cell count (cells/mm^3^). The x-axis shows the time in years since the HIV-2 diagnosis. Line through the data is a simple linear regression, and data shown are the slope and 95% CI. All participants were elite controllers with HIV-2 plasma viral RNA <200 copies/mL with only one blip >200. Additionally, these participants were in care for >10 years. *A*, Five participants had an increase in CD4+ T-cell count after diagnosis. *B*, Five participants had a decline of CD4+ T-cell count after diagnosis.

Thirteen people with HIV-2 in this cohort were ECs. This group was almost exclusively composed of women (92.3%; Table 2). Median age at diagnosis was 36 years and 12 ECs were born in West Africa. All ECs presented with a CDC category A (except 1 unknown) and had a mean CD4+ T-cell count of 750 cells/mm^3^ (IQR, 670–850) at diagnosis. Three ECs were HLA-B57 negative (23.1%); for the other ECs, no HLA typing was known. Seven ECs remained undetectable at every plasma HIV-2 RNA measurement. None had a hepatitis B or C coinfection. Three women did use ART during pregnancy: 1 woman for 1 pregnancy, 1 for 2 pregnancies, and 1 for 3 pregnancies. All of them discontinued ART postpartum. Ten ECs had a follow-up >10 years. When we looked at the CD4+ T-cell counts of these 10 ECs, 5 had an increase in CD4+ T-cell count (median follow-up, 16 years; Figure 1*A*). These ECs had a median age of 37 years at diagnosis (IQR, 36–37). Four others had decreasing CD4+ T-cell counts over time, with 1 participant remaining relatively stable (median follow-up, 20 years; Figure 1*B*). These 5 ECs had a median age of 33 years at presentation (IQR, 25–40), and only 1 participant was older than 50 years at the time of diagnosis. Plasma HIV-2 blips of the 13 ECs are shown in Supplementary Table 1.

### Recontroller

**Figure 2.**
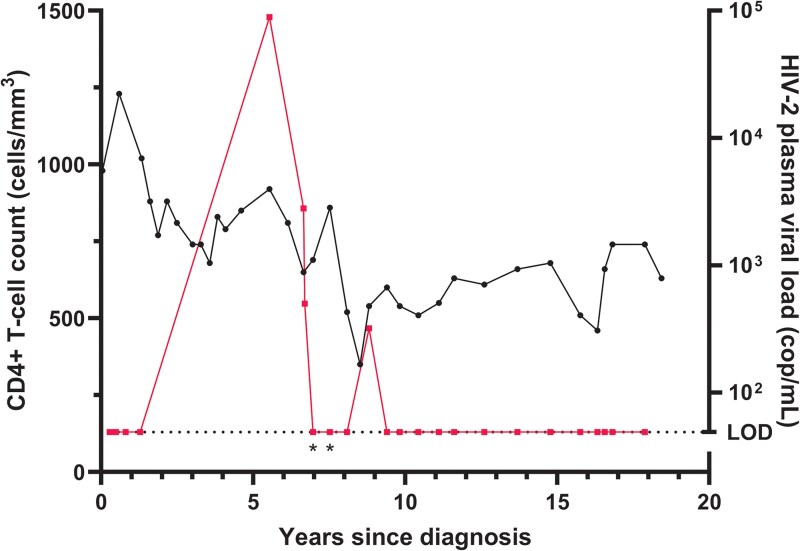
A person with spontaneous resuppression without the use of antiretroviral therapy after HIV-2 reactivation. Left y-axis shows the CD4+ T-cell count (cells/mm^3^) with corresponding data in black (circles). The right y-axis shows the HIV-2 plasma viral load (copies/mL) with corresponding data in pink (squares). The x-axis shows the time in years since HIV-2 diagnosis. The dotted line depicts the limit of detection (LOD) of the HIV-2 viral load assay (50 copies/mL) with an asterisk if the assay limit of detection was 500 copies/mL.

One person showed an interesting pattern of viral loss of control and recontrol. This participant was diagnosed with a CD4+ T-cell count of 980 cells/mm^3^ and an undetectable viral load. He maintained an undetectable HIV-2 plasma viral load with CD4+ T-cell counts ranging from 680 to 1230 cells/mm^3^ (Figure 2). Approximately 5 years after diagnosis, a peak in plasma viremia occurred (8.9 × 10^4^ copies/mL). The participant did not start ART due to a high CD4+ T-cell count, following the guidelines in place at that time. Within 2 years, this person was able to control plasma viremia. Two years after control, another, albeit smaller, peak occurred (320 copies/mL), which the participant was also able to control. Just prior to this peak, the participant dipped to 350 CD4+ T cells/mm^3^. Undetectable plasma HIV-2 RNA was maintained for >10 additional years. This participant could qualify as an EC for the latter part of the observed HIV-2 infection.

### Loss of Control

**Figure 3.**
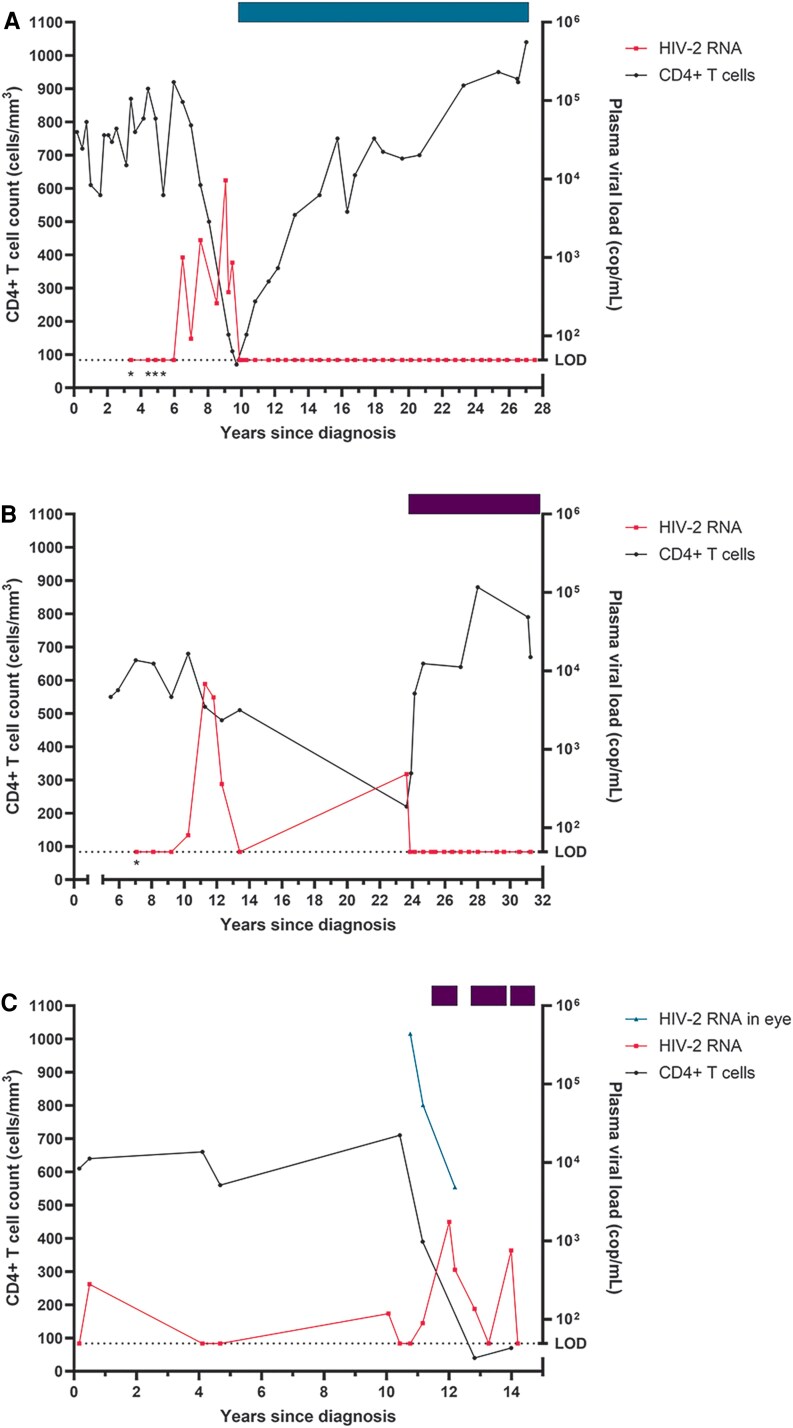
CD4+ T-cell counts and plasma viral loads for participants with loss of viral control. Left y-axis shows the CD4+ T-cell count (cells/mm^3^) with corresponding data in black (circles). Right y-axis shows the plasma viral load for HIV-2 (pink, squares) and HIV-2 in aqueous humor if applicable (blue, triangles). The x-axis shows the time in years since HIV-2 diagnosis. The dotted line depicts the limit of detection (LOD) of the HIV-2 viral load assay (50 copies/mL) with an asterisk if the assay limit of detection was 500 copies/mL. The bar on top of the figure shows the antiretroviral therapy regimens. Purple: 3TC + ABC + DTG (*B* and *C*). Blue: 2× NRTI + boosted PI (*A*), 9.9–10.5 years; 3TC + AZT + LPV/r, 10.5–22.5 years; LPV/r + ABC + 3TC, from 22.5 years; ABC + 3TC + DRV/c. Abbreviations: 3TC, lamivudine; ABC, abacavir; AZT, zidovudine; DRV/c, darunavir-cobicistat; DTG, dolutegravir; LPV/r, lopinavir-ritonavir; NRTI, nucleoside reverse transcriptase inhibitor; PI, protease inhibitor.

We encountered 3 people who started as ECs but lost viral control, switching to viremic progressors (Figure 3). All 3 persons presented with no HIV- or AIDS-defining conditions or other symptoms and a median CD4+ T-cell count of 690 cells/mm^3^ (IQR, 650–730; Table 2). After loss of control, HIV RNA zenith was a median 6800 copies/mL and all initiated ART. One participant was particularly interesting as this person maintained CD4+ T-cell counts between 560 and 710 cells/mm^3^ and a viral load <300 copies/mL with mostly undetectable viral loads for >10 years after diagnosis (Figure 3*C*). The participant then presented at the clinic with tingling sensations in the eye and reduced sight. HIV-2 was detected in the aqueous humor of the anterior chamber at a concentration of 4.39 × 10^5^ copies/mL while HIV-2 plasma viral load was undetectable. CD4+ T-cell counts decreased to 390 cells/mm^3^. After initiation of ART, the viral load in the eye decreased more than almost 10-fold; however, a temporary increase in viral load in the blood was seen to 1750 copies/mL, possibly due to low adherence. Viral load remained uncontrolled except at 2 visits, and CD4+ T-cell counts stayed low. Unfortunately, the patient was lost to follow-up.

### Nonviremic Progressor

**Figure 4.**
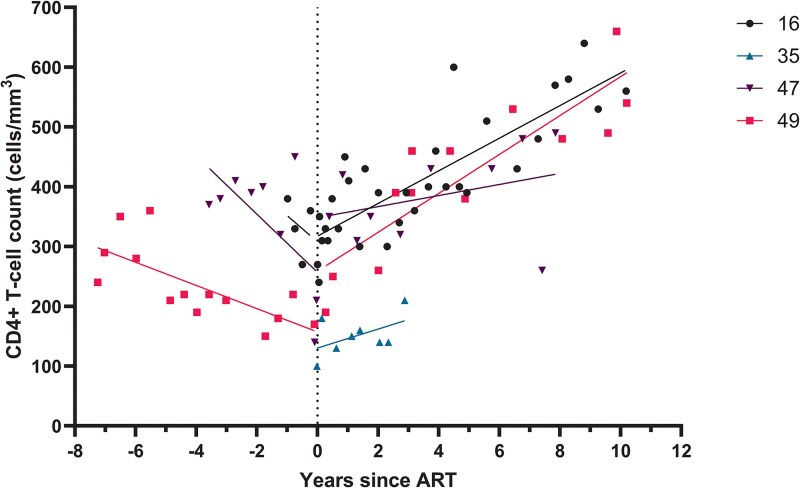
CD4+ T-cell counts in HIV-2 nonviremic progressors. The y-axis shows the CD4+ T-cell count (cells/mm^3^). The x-axis shows the time in years before or after start of ART. The line through the data points is a simple linear regression of data points before and after the start of ART. Abbreviation: ART, antiretroviral therapy.

Four participants in the cohort initiated ART because CD4+ T-cell counts were or had decreased to <350 cells/mm^3^, while 3 of 4 had an undetectable viral load at all measurements and 1 had only a single measurable viral load of 71 copies/mL (Table 2). We named this category nonviremic progressors. One nonviremic progressor was negative for HLA-B57 while another was positive for HLA-B57; for the 2 other participants, HLA typing was unknown. All nonviremic progressors presented with CDC stage A, although CD4+ T-cell counts at diagnosis were low, with a median 305 cells/mm^3^ (IQR, 205–373). All 4 nonviremic progressors initiated ART without any HIV-related symptoms being present, after which there was a marked increase in CD4+ T-cell count (Figure 4).

### Viremic Progressors

The largest category in this cohort consisted of viremic progressors (n = 19). These participants had high HIV-2 viral loads and decreasing or low CD4+ T-cell counts, indicating no level of control. All viremic progressors initiated ART. Viremic progressors were born in West Africa or Europe and diagnosed in CDC stages A through C (Table 2). Of the 6 viremic progressors who presented at CDC stage C, 4 presented with *Pneumocystis jirovecii* pneumonia and 1 with HIV nephropathy, whereas 1 had cytomegalovirus encephalitis, hepatitis, colitis, and retinitis. These viremic progressors had the highest viral load zenith at a median 4.75 × 10^5^ copies/mL (IQR, 10 875–112 250) as compared with other categories and presented with a median CD4+ T-cell count of 110 cells/mm^3^ (IQR, 70–190). One died because of invasive aspergillosis and neutropenia with possible T-cell lymphoma, and the other died from an extensive cytomegalovirus infection; thus, both deaths were classified as HIV/AIDS related.

### HIV-1 and HIV-2 Coinfections

Five participants had confirmed coinfection with HIV-1 and HIV-2. These people had extremely low CD4+ T-cell counts at diagnosis (median, 50 cells/mm^3^) and all initiated ART.

### Pre-ART

Seven participants died before 1997, before combination ART was available. These participants had a median age of 35 years at presentation, and all died due to AIDS within 5 years of diagnosis at a median age of 36 years. If cause of death was registered, it included Kaposi sarcoma, tuberculosis, toxoplasmosis, wasting, HIV nephropathy, *Candida* esophagitis, and cryptococcosis.

## DISCUSSION

Previous research on HIV-2 has classified individuals as either controllers or progressors. However, the long follow-up in our cohort with HIV-2 allowed us to define several levels of control. Control was identified by the examination of clinical trajectories, HIV-2 plasma viral load, and CD4+ T-cell counts. For HIV-1, there is a substantial heterogeneity and overlap in the definitions used to classify phenotypes of HIV-1 control and progression, which hampers uniform interpretation of existing data [19–21]. In addition, definitions used in HIV-1 may not be directly applicable to HIV-2. For example, nonviremic progressors are a phenotype observed in HIV-2 but not HIV-1. Moreover, no uniform thresholds for plasma HIV-1 viral load and CD4+ T-cell counts are used to classify the HIV-1 phenotypes, although HIV-1 viral load <50 copies/mL and/or CD4+ T-cell count >500 cells/mm^3^ is frequently used to define long-term nonprogressors and ECs [19]. In our study, we used a plasma HIV-2 viral load threshold of 200 copies/mL and a CD4+ T-cell count threshold of 350 cells/mm^3^. These thresholds were based on our clinical experience with HIV-2. For example, person 14 is an HIV-2 EC with CD4+ T-cells counts between 350 and 500 cells/mm^3^ at diagnosis and a subsequent durable increase to >500 cells/mm^3^. Person 8 is also an HIV-2 EC with CD4+ T-cell counts always >500 cells/mm^3^ despite multiple blips of HIV-2 plasma viral load between 50 and 200 copies/mL. It is notable that mean CD4:CD8 ratios at diagnosis seemingly correlated with the clinical outcome, raising the possibility that this parameter may be predictive of EC status. Yet, these results may be biased by time of diagnostics, and the small number of participants limited our ability to test the predictive value of this parameter.

We identified 13 participants in our cohort as ECs, with the ability to suppress HIV-2 to levels <200 copies/mL and retain CD4+ T-cell counts well above 350 cells/mm^3^ without the use of ART. Within the ECs that we followed for over a decade, we observed 2 subcategories: 1 group of participants exhibited an increase in CD4+ T-cell counts over time, while the other group experienced a slow decline, albeit without any known clinical consequences to date. Another category of participants exhibited an EC phenotype for at least 5 years but then unexpectedly lost control. One person also lost control but regained the ability to suppress plasma viremia to undetectable levels for almost a decade, without the use of ART, and could be considered an EC. A fourth category, nonviremic progressors, had progressive CD4+ T-cell counts decline until <350 cells/mm^3^ despite undetectable viral loads. Initiation of ART resulted in increasing CD4+ T-cell counts. We also report a category of viremic progressors that exhibited a classical phenotype of HIV—low CD4+ T-cell counts with high HIV-2 plasma viral loads—and a small category of participants who died at a young age before ART was available. These and HIV-1/2 double infections are not discussed here. We think that understanding these patterns of control and loss or gain of control provides valuable insights into HIV-2 disease progression, which can guide medical decisions regarding ART use and in research toward a functional cure for HIV.

Two types of HIV-1 controllers have been described: ECs as defined in this article and long-term nonprogressors who have detectable viral loads but stable CD4+ T-cell counts [22]. Interestingly, no long-term nonprogressor phenotype was found in our study, suggesting that the presence of HIV-2 plasma viremia may be of more impact on the immune system than the presence of HIV-1 plasma viremia. Elite control in HIV-1 is determined by a combination of genetic factors, immune responses, and viral characteristics, but data on HIV-2 elite control are limited [3, 23, 24]. Considering that 12 of 13 ECs in our cohort were women, this could indicate a genetic component. More ECs originated from West Africa than from other areas, which could suggest a role for HLA involvement. For HIV-1, a correlation with HLA B*57 and EC has clearly been shown [25]. For HIV-2, no HLA B*57 epitopes have been identified, although 1 report mentioned cross-presentation of epitopes by B*57 and B*58 [26]. Our cohort had minimal data on HLA-B57, leaving its role in control undetermined; however, the prevalence of B*57 and B*58 in Cape Verde is low (1.6% and 1.6%–4%, respectively) [27]. Given the higher prevalence of ECs among people with HIV-2, viral factors appear to be particularly significant. Previous research in our laboratory, performed with viruses from this cohort, investigated some viral characteristics of HIV-2. A viral outgrowth assay revealed a lower infectious load in participants with an undetectable viral load as compared with long-term nonprogressors with HIV-1, and HIV-2 had lower replication rates [17, 28]. Yet, in a previous study, we analyzed near full-length genome sequences in some of this cohort [5]. With phylogenetic analysis, we identified genotypic markers associated with disease progression. Our findings did not reveal any genotypic defects that could explain HIV-2 nonprogression. Recent data on integration sites in HIV-1 ECs saw that intact proviruses were more often found in heterochromatin regions, while defective proviruses were more often located in permissive genic euchromatin positions [29]. This suggests immune pressure on intact proviruses, although no data yet exist showing this in ECs in HIV-2. It remains unclear whether this significantly differs from HIV-1 and how it varies between ECs and non-ECs. More research needs to be done in ECs in HIV-2 to be able to explain the differences in prevalence and mechanisms of viral control.

We identified a category of people with HIV-2 who initially demonstrated the ability to suppress the virus but abruptly lost this control in terms of viral control and immunologically and therefore were prescribed ART. It could be that the viremic progressors identified in our cohort also belong to the category of loss of control, but they were not diagnosed at that stage. Similarly, the ECs identified in our cohort may lose virologic and immunologic control somewhere in the future. This shows that lifelong clinical, virologic, and immunologic follow-up is required for people with HIV-2 because their clinical phenotypes may change over time. The loss-of-control phenotype has been reported in HIV-2 as well as HIV-1 [22, 30, 31]. In terms of possible causes for loss of control, these have been more extensively detailed in HIV-1. There have been case reports of ECs with HIV-1 that have a superinfection with another HIV-1 virus, causing viral rebound [32]. We did not exclude the possibility of superinfection in our study. Another potential explanation for loss of control could be tropism switch from CCR5 to CXCR4 [33]. Our previous research has shown that within this cohort, HIV-2 replication rates were increased in people who progress to AIDS as compared with ECs, and replication rates also increase with the switch from CCR5 to CXCR4 [28]. In these participants, we did not determine whether there was coreceptor switching, but there was already broad coreceptor usage observed in viruses from aviremic and viremic cases in a cross-sectional study [15]. Especially in the case of the recontroller, this hypothesis is less likely. Rather, a transient decrease in CD4+ T-cell counts caused by another infectious agent, comedication, or another event may have triggered loss of immunologic control before immune restoration controlled viral loads.

We observed a phenotype in participants who, despite having no detectable viral load without ART, had significantly declining CD4+ T-cell counts: the nonviremic progressors. Upon initiation of ART, their CD4+ T-cell counts increased. While mentioned in literature, this phenotype has not been described in detail. Recent research in women in Senegal who were not undergoing ART and had high CD4+ T-cell counts revealed that the HIV-2 proviral landscape is predominantly composed of defective proviruses [34]. This is similar to HIV-1, and it has been shown that defective proviral HIV-1 can still produce viral proteins recognizable by cytotoxic T cells. This could lead to increased cell death, immune activation, and immune exhaustion, which in turn could lead to a decrease in CD4+ T cells [34, 35]. Additionally, considering the loss of viral control in the single recontroller, CD4+ T-cell counts decreased before viral loads increased. Alternatively, the gradual decline in CD4+ T-cell counts observed in some may reflect replication in tissues not detectable in plasma. Many people with HIV are currently prescribed ART, irrespective of viral load and CD4+ T-cell count, and this might also benefit some ECs with HIV-1 [36]. Given our data and the data demonstrating that people with HIV-2 develop AIDS at higher CD4+ T-cell counts, we recommend close monitoring of CD4+ T-cell counts and clinical events indicating progressive HIV-2 disease in ECs with HIV-2 who are not on therapy [37]. Special attention should be given to the category of ECs who exhibit a gradual clinically significant decrease in CD4+ T cells, where initiating ART should definitely be considered to prevent AIDS-related illness.

A limitation of our study is that the lower detection limit of the viral load assay changed during the long follow-up. We defined an EC as an individual with a viral load <200 copies/mL, while at some time points we have data only with the test detection limit at 500 copies/mL. There is a possibility that ECs may have had a viral load between 200 and 500 copies/mL before the standard test switch. However, long follow-up and viral loads <50 copies/mL confirmed that these individuals were genuine ECs. Additionally, our cohort of people with HIV-2 has a limited size and is a purely retrospective descriptive study with no extensive immunologic analysis. Yet, a strength of this cohort is that our follow-up is extensive, with a median duration of 16 years. This cohort of people with HIV-2 is among those with the longest follow-up periods, and this extensive follow-up time allowed us to describe and define different levels of control. We hope that these data and specific groups can be validated in other cohorts of people with HIV-2.

Our findings reveal a more complex picture of viral control in HIV-2 than previously proposed. We classified participants based on their clinical phenotype, leading to the discovery of several distinct categories, some of which have not yet been extensively described for HIV-2. The most relevant of which may be nonviremic progressors: people who can control HIV-2 viral replication but nevertheless had relevantly decreasing CD4+ T-cell counts, which increased significantly upon starting ART. This highlights the necessity to continue monitoring ECs and start ART before AIDS can occur. Future research should also study the differences among the different levels of control to uncover potential biomarkers or specific viral or immunologic characteristics that might explain their ability to control the virus. By understanding these differences, we hope to make significant strides toward optimal treatment and finding a cure for all people with HIV.

## Supplementary Material

ofaf336_Supplementary_Data

## Supplementary Data

Supplementary materials are available at *Open Forum Infectious Diseases* online. Consisting of data provided by the authors to benefit the reader, the posted materials are not copyedited and are the sole responsibility of the authors, so questions or comments should be addressed to the corresponding author.
