# Supplementary material for: Insights to a Cure: Unique Controller Phenotypes in the Rotterdam HIV-2 Cohort
Source: Open Forum Infect Dis. 2025 Jun 18;12(7):ofaf336. doi: 10.1093/ofid/ofaf336 (PMC12207973; doi:10.1093/ofid/ofaf336)
Supplement: ofaf336_Supplementary_Data [file ofaf336_supplementary_data.docx]

**Supplementary Table 1: Plasma HIV-2 blips in elite controllers.**

| **#** | **Plasma HIV-2 blips** | **Follow-up time (years)** |
| --- | --- | --- |
| **3** | 1: 57 copies/mL | 31 |
| **8** | 9 blips <200 cop/mL | 29 |
| **12** | none | 2 |
| **14** | none | 19 |
| **22** | 1: 371 copies/mL | 16 |
| **34** | 1: 680 copies/mL | 20 |
| **40** | none | 19 |
| **41** | 1: 87 cop/mL  2: 77 cop/mL  3: 102 cop/mL | 17 |
| **42** | none | 23 |
| **43** | none | 16 |
| **44** | none | 14 |
| **48** | none | 4 |
| **52** | 1: 89 copies/mL  2: 81 copies/mL | 2 |
